# Heavy Metal Accumulation in Water, Soil, and Plants of Municipal Solid Waste Landfill in Vientiane, Laos

**DOI:** 10.3390/ijerph16010022

**Published:** 2018-12-21

**Authors:** Noudeng Vongdala, Hoang-Dung Tran, Tran Dang Xuan, Rolf Teschke, Tran Dang Khanh

**Affiliations:** 1 Graduate school for International Development and Cooperation, Hiroshima University, Hiroshima 739-8529, Japan; vongdala1986@gmail.com; 2 Nguyen Tat Thanh University, Ho Chi Minh City 702000, Vietnam; tranhoangdung1975@yahoo.com; 3 Department of Internal Medicine II, Division of Gastroenterology and Hepatology, Klinikum Hanau, D-63450 Hanau, Germany; rolf.teschke@gmx.de; 4 Agricultural Genetics Institute, Pham Van Dong, Tu Liem, Hanoi 123000, Vietnam; tdkhanh@vaas.vn

**Keywords:** municipal solid waste, landfill, heavy metals, soil, plants, water, pollution, health risk

## Abstract

The municipal solid waste (MSW) landfill in Vientiane, Laos, which receives > 300 tons of waste daily, of which approximately 50% is organic matter, has caused serious environmental problems. This study was conducted to investigate the accumulated levels of heavy metals (HMs) (cadmium (Cd), chromium (Cr), copper (Cu), nickel (Ni), lead (Pb), and zinc (Zn)) in water (surface and groundwater), soil, and plants between dry and wet seasons according to the standards of the Agreement on the National Environmental Standards of Laos (ANESs), Dutch Pollutant Standards (DPSs), and the World Health Organization (WHO), respectively. Although no impact of pollution on the surface water was observed, the levels of Cr and Pb in the groundwater significantly exceeded the basics of ANESs and WHO in both seasons. The pollution caused by Cd and Cu reached the eco-toxicological risk level in the landfill soils and its vicinity. The vegetable *Ipomoea aquatica*, which is consumed by the nearby villagers, was seriously contaminated by Cr, Pb, Cu, and Zn, as the accumulation of these toxic metals was elevated to much greater levels as compared to the WHO standards. For the grass *Pennisetum purpureum* (elephant grass), the quantities of HMs in all plant parts were extreme, perhaps due to the deeper growth of its rhizome than *I. aquatica*. This study is the first to warn of serious HM pollution occurring in the water, soil, and plants in the MSW landfill of Vientiane, Laos, which requires urgent phytoremediation. The indication of what sources from the MSW principally cause the pollution of HMs is needed to help reduce the toxicological risks on Lao residents and the environment in Vientiane as well.

## 1. Introduction

The management of municipal solid waste (MSW) is a challenge for the urban environment in many developing countries and is attributed to rapid population growth, unsatisfactory urbanisation, and undesireable economic growth. Thus, open dumping and unsanitary waste landfills are a pressing issue. MSW comprises household, healthcare, and industrial waste, but they are not segregated and are all disposed of into the same landfill [1]. The landfill is the principal place for solid waste dumping, which has resulted in serious environmental pollution and the spread of disease [2,3]. Leachate migration in open dumping sites is a dominant source of heavy metals (HMs) in surface and groundwater, soil, and plants [4,5,6]. If plants uptake HMs from polluted soil, there is a high possibility of HMs transferring to the human food chain through the consumption of vegetation or animals [7,8]. The most problematic HMs include cadmium (Cd), chromium (Cr), copper (Cu), lead (Pb), nickel (Ni), and zinc (Zn) [9,10]. Contaminated wastewater has resulted in the contamination of food crops [11,12]. 

When wastewater contains HMs at low concentrations, they may be useful for increasing productivity in agricultural production, as they are essential to living organism growth. However, high concentrations of HMs have negative effects on the environment [13,14]. Many studies have documented that both individual and combined HMs simultaneously expose humans and other organisms to toxic effects but are mediated by leached doses, duration of exposure, and genetic factors [15]. There are many compounds with hazardous effects leaching from MSW landfills that can harmfully influence human health and the environment. In addition, several hazardous compounds have also been found in the sediments but not in leachates. However, the leachates from MSW are a fundamental matrix of toxic contaminants to freshwater aquatic organisms [16,17].

Plants growing in an MSW landfill and its vicinity cycle both trace elements and contaminated HMs that can affect the food chain and can accumulate trace elements, especially HMs. They receive HMs from soils and partly from water and air [18]. However, some plants, such as *Myriophylhum aquaticum* (parrot feather), *Ludwigina palustris* (creeping primrose), and *Mentha aquatic* (water mint), have shown excellent phytoremediation strength for contaminated soils, groundwater, and wastewater by absorption [19,20]. In addition, the phytoremediation from plants is an efficient treatment technique for HMs due to its low cost and easy operation and maintainance [21,22]. 

The MSW landfill in Vientiane, Laos receives around 300 tons of waste daily, of which 40–50% is organic matter. The sources of solid waste include residential, commercial, institutional, and industrial activities [23]. When hazardous waste from hospitals and industry is disposed of into the landfill, it causes groundwater and surface water pollution [24]. In the wet season, surface water runs off from the landfill [25]. Therefore, analyses for HMs should be conducted on samples collected during both the dry and wet seasons. Residents in areas close to the MSW landfill who work at the dump, such as waste pickers, may have serious health problems. However, an analysis of chemical leachates in soils, water, and plants growing in the MSW has not yet been evaluated. This research was thus conducted to assess HM concentrations in water, soil, and plants in the landfill and surrounding area to understand the pollution situation and to help establish an appropriate management strategy. In addition, this information can help develop policies for site remediation and urban environmental quality management.

## 2. Materials and Methods

### 2.1. Description of Research Area

The MSW landfill is located in a suburban area of Vientiane, the capital of Laos (latitude 18°4′45.86″ N, longitude 102°50′57.18″ E, approximately 32 km from the urban centre). The landfill operation is open dumping in a total area of 100 ha of land space and has been operating since 2007. The area has a tropical climate characterized by wet season rainfall from May to September and a dry season from October to April. The MSW landfill is a flat field adjacent to agricultural land, including rice fields. It includes landfill management offices, a recycling factory, wastewater treatment ponds, wetlands, and a temporary hut for waste pickers. Residents in the surrounding areas of the MSW landfill have never received public health information. Many plants in the landfill such as *Ipomoea aquatica* (water spinach) are collected and sold in the nearby local markets for human consumption. The landfill location is also connected to small substreams, and wastewater runoff in the rainy season overflows to the rivers. Also, the residents catch fish from the polluted ponds and nearby streams, which are sold to local markets without concern for possible HM pollution from MSW.

**Figure 1:**
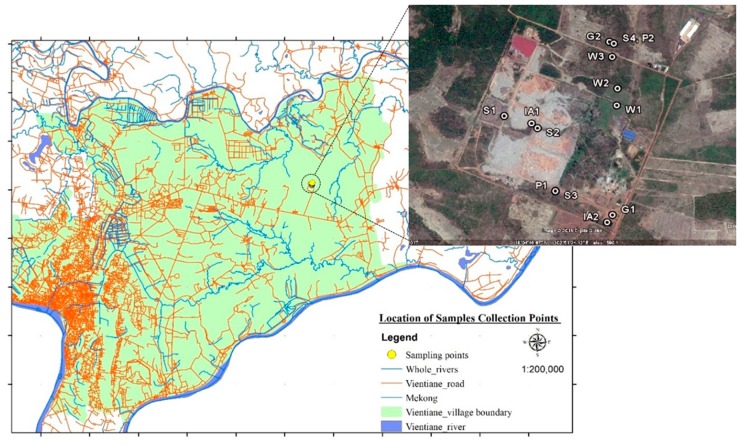
Map and satellite image of sampling locations in the landfill site.

In this study, samples from surface water were collected upstream to downstream from wetlands in the landfill site (Table 1). Groundwater and soil samples were gathered from inside and outside of the landfill. In addition, two plant species—*I. aquatica* and the grass *Pennisetum purpureum* (elephant grass)—were also collected from the landfill and surrounding area (see Figure 1 or Table 1).

### 2.2. Sample Collection

Three samples from the surface water in the landfill wetlands, from an average water depth of 1–1.5 m, were collected between each station from upstream to downstream. Good quality samples were randomly taken each season, and each station included two subsamples. They were stored in 1-L polyethylene bottles and subsequently adjusted by HNO_3_ to obtain pH < 2 [26,27]. The values of pH, temperature (°C), electrical conductivity (EC), and dissolved oxygen (DO) were measured by HORIBA (U-50 Multiparameter Water Quality Meter, Kyoto, Japan) at field sites. Furthermore, two groundwater samples were collected from wells inside and in the vicinity of the landfill. The G1 (well) was used for the landfill management office at the landfill, and the G2 (well) was far from the landfill (70 m away) and it was frequently exploited by nearby farmers. The characterized flow of groundwater (G2) was unknown. The water samples were taken in both wet and dry seasons in 2017, and they were preserved in a way similar to the surface water samples. In detail, pH values of 8.32 and 7.53, temperatures of 19.14 and 25 °C, DO values of 12.03 and 0.52 mg/L, and EC values of 2.77 and 6.93 µs/cm were recorded in wet and dry seasons, respectively. The results showed that these parameters fitted the standards of the permissible limits of the National Environmental Standards of Laos, except the DO in the dry season.

Soil samples were randomly collected at 0–0.25-m depth at an area of 50 cm^2^ in the landfill and locations at 60 m from the landfill according to a method described in Gworek et al. [28]. There were 48 samples gathered in both wet and dry seasons in 2017. The locations of soil samples inside the dumpsite were collected from the nearest recharge canals. At each sampling location, six subsamples were randomly collected to make a composite sample. The soils were placed in sterilized plastic bags, while the sampled sites were recorded by GPS (GARMIN GPS 62_sc_ (surface and groundwater, soil and plant samples) (Table 1). The soils were air-dried at room temperature after collection. They were finely ground by an agate mortar, filtered through a 0.2-mm stainless-steel sieve to remove coarse debris, and stored in sealed containers at 4 °C for further analysis.

*I. aquatica* (water spinach) was collected in two ponds in the landfill, including leachate and fish ponds. The samples were collected in both wet and dry seasons (total of 24 samples). The roots, stems, and leaves were separated and stored in zipped polyethylene bags. The samples were washed thoroughly with tap water and rinsed with distilled water for 1 min to clear them of periphyton and detritus [28]. The *P. purpureum* samples were collected in two locations: inside and outside the landfill. The samples were dried at 40 °C for 2 days in an oven until a consistent weight was maintaned. The samples were ground into a fine powder by a mortar and kept in the dark at 5 °C for further analysis.

### 2.3. Chemical Analysis for Heavy Metals

The surface and groundwater samples were filtered by filter papers (Qualitative, circle, 90 mm Ø, Whatman^TM,^ New Jersey, US) to obtain a 100-mL solution, to which 1 mL of HNO_3_ (65%) was added and then heated for 2 h without boiling at 80–90 ℃. The samples were cooled to room temperature and then filtered with 0.2-μm syringe filters [29]. The waters were analyzed using a multitype inductively coupled plasma emission spectrometer (ICP-ES) and ICPE-9000 (Shimadzu, Tokyo, Japan).

Five grams of soil were added to 20 mL of HNO_3_ (7 mol/L), stirred for 1 h, and then placed in an autoclave at 120 °C for 30 min. The mixture was cooled to room temperature, filtered by filter papers, and diluted by deionized water [30]. The samples were filtered again by a 0.2-μm syringe filter and transferred to tubes for analyses by ICPE-9000 (Shimadzu, Tokyo, Japan).

The roots, stems, and leaves of the plant samples were dried and ground into powder. An amount of 0.5 g of each sample was added to 6 mL of concentrated HNO_3_ (65%) and 2 mL of concentrated HCl (30%) and stood until the reaction was completed. The mixture was moved to an autoclave for 66 min at 132 °C for digestion [30]. The plant samples were analyzed by ICPE-9000 (Shimadzu, Tokyo, Japan).

### 2.4. Statistical Analysis

The statistical analyses were implemented by Minitab^®^ 17.3.0 (Copyright © 2016 by Minitab Inc., Shanghai, China). Mean and standard deviation values were evaluated using one-way analysis of variance (ANOVA). Tukey’s test was applied to compare pairs among seasons, treatments, and data of the standards at a *p*-value of *p* < 0.05, which indicated the significant differences by 95% confidence.

## 3. Results

### 3.1. Concentration of Heavy Metals in Surface and Groundwater

**Figure 2:**
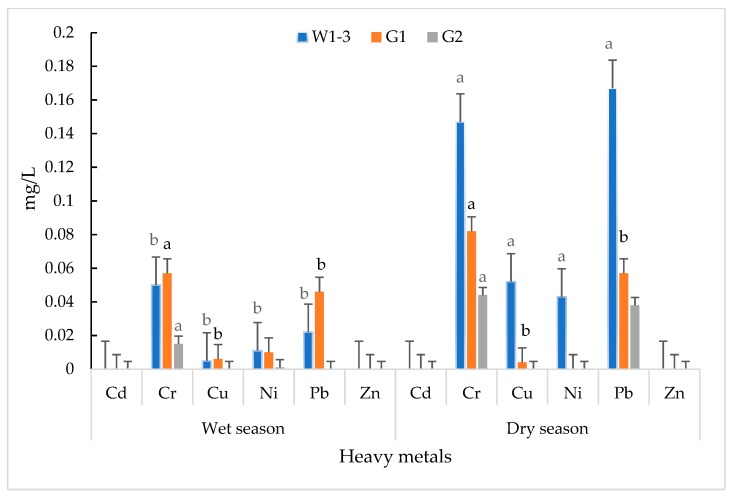
Seasonal variation of heavy metals in water. Bar values are mean ± standard error (SE); bars with similar letters are not significantly different (*p* < 0.05).

Table 2 shows that several HM contents were below the detectable limit of the analyzed instrument and were noted as not detected (ND). The results in Table 2 indicate that no trace of cadmium (Cd) and zinc (Zn) was detectable in surface and groundwater of both the wet and dry seasons. The accumulation of chromium (Cr), copper (Cu), nickel (Ni), and lead (Pb) was observed, but none of them exceeded the standard levels of the Agreement on the National Environmental Standards of Laos (ANESs). Similarly, no presence of Cd and Zn was found in G1 (groundwater inside the landfill) and G2 (groundwater outside the landfill) in both the wet and dry seasons (Table 2; Figure 2). However, Ni was not detected in both G1 and G2 in the dry season, whilst Pb was not found in the wet season in G2. In addition, no accumulation of Cu was observed in G2 in both the wet and dry seasons (Table 2; Figure 2). In the groundwater inside the MSW landfill, the levels of Cr and Pb significantly exceeded the standards of both ANESs and the World Health Organization (WHO). On the other hand, traces of Ni and Cu were below the permissible limit of ANESs and WHO (Table 2). In locations outside the landfill (G2), although Cr, Ni, and Pb were detected, only the contents of Pb in the dry season were much higher than those of ANESs and WHO standards (Table 2). The levels of HMs in the dry season were higher than the wet season. The results suggest that seasonal variation significantly influences heavy metal concentrations.

### 3.2. Concentration of Heavy Metals in Soil

**Figure 3:**
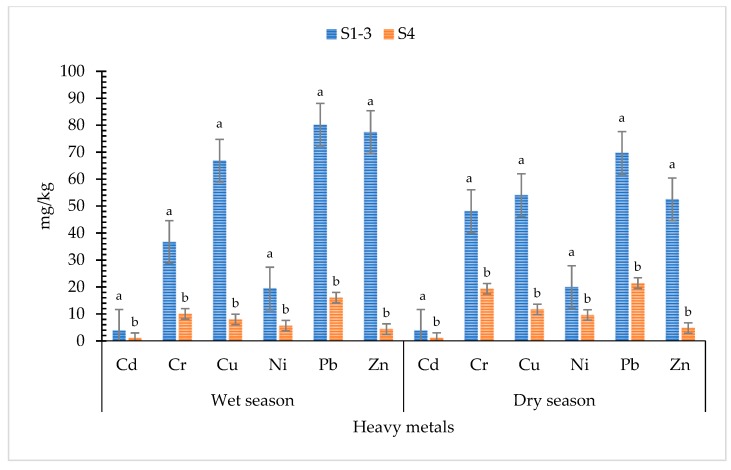
Significant difference in two locations and seasonal variation of heavy metals in soils. Bar values are mean ± SE; bars with similar letters are not significantly different (*p* < 0.05).

Table 3 shows that the HMs from soils in the landfill were generally in much greater quantities than those of soils from the nearby location of the MSW landfill. The amount of HMs differed, and Zn was the greatest (>17-fold), whilst Cd and Cr were the least (>3-fold) (Figure 3). On the other hand, the quantities between the wet and dry seasons varied among the HMs. Amounts of Cd and Cu in the landfill exceeded the target value of the Dutch Pollutant Standards. In addition, the level of Cd overcame the eco-toxicological risk (outside landfill) (Table 3).

### 3.3. Concentrations of Heavy Metals in Plants

Table 4 shows that the roots of *I. aquatica* accumulated higher quantities of HMs than those allowed by WHO standards in both the wet and dry seasons. The maximal pollution levels were recorded for Cd, Cr, Pb, and Zn (8–56-fold higher than the WHO standards) in the stems and roots (Table 4). The leaves of this vegetable were not affected by Cd, Cu, Ni, Cd, and Ni in the wet and dry seasons, respectively, whilst the HM levels in stems did not exceed the standards of WHO in Cd, Cu, and Ni in the wet and dry seasons, respectively (Table 4). Considering that the villagers commonly consume leaves and stems of *I. aquatica*, Cr, Pb, Cu, and Zn in this vegetable presented in much higher quantities than the WHO standards (Table 4).

For *P. purpureum*, the quantity of Cd in its roots was > 50-fold greater than that of *I. aquatica* in the landfill, although the accumulated level of Cd was > 8-fold lower than the plants growing outside the MSW (Table 4). In the dry season, the amount of Cd in the rhizome of *P. purpureum* was also reduced by > 4-fold compared to the wet season. The accumulation of Cd in the stems and leaves of *P. purpureum* was similar to its roots. This evidence indicates that in the wet season, *P. purpureum* absorbs a much greater amount of Cd than in the dry season (Table 4). Furthermore, Cr, Cu, Ni, Pb, and Zn in different plant parts of *P. purpureum* were analogous to that of Cd. These chemicals were accreted in much higher quantities than that of *I. aquatica* and all of them exceeded the WHO standards, except for Ni in the leaves of this grass (Table 4). The accumulation of these HMs was significantly reduced in the dry season. Except for Ni, the presence of Cd, Cr, Cu, Pb, and Zn was found in all plant parts of *P. purpureum* growing outside the landfill in the dry season. This revealed that the pollution caused by Ni was less problematic than other HMs (Table 4). The amount of HMs detected in *P. purpureum* may be explained by the deeper roots of the grass, as *I. aquatica* roots commonly spread either on the surface of the soil or water in the landfill.

## 4. Discussion

In this study, the surface water showed values lower than the standards (data not shown); therefore, it did not affect the environment. In contrast, the groundwater in the wet and dry seasons had high concentrations of Pb and Cr that exceeded the permissible values of ANESs [31] and WHO [32,33]. High doses of Pb and Cr detected in groundwater might be due to the leachate of HM contamination from MSW [34,35]. However, the amounts of Cd, Cu, Ni, and Zn in both seasons were lower than the permissible value of the regulations [31,32]. Cd and Zn in the groundwater were not detected, perhaps because of the low content of Cd and Zn in the MSW [36], which might be reduced by plant absorption [37].

In general, the dry season showed higher HM concentrations than the wet season. Probably, in the wet season, low-strength leachate was generated; however, during the dry season, the reduced percolation and enhanced evaporation might have increased the leachate strength [38]. Although the accumulation of Cd, Cu, Ni, and Zn was lower than Pb and Cr in the groundwater (Table 2), it was reported that the long-term oxidation of residual organic matters, together with sulfur, nitrogen, and iron in MSW, may lead to the release of greater amounts of HMs [39].

The Cd and Cu in the soil samples exceeded the levels of Dutch Pollutant Standards to reach an eco-toxicological risk [34]. The higher amount of Cd than the target value [34] in each station might result in broader contamination by leachate migration. Vegetation that absorbs an excessive amount of Cd and other HMs (Table 4) may adversely influence the health of people and animals living in the landfill and in its vicinity [40]. The tremendous amount of Cd and Cu may be related to the high quantities of these toxic metals in waste compositions that are disposed of in the landfill [41]. Kitchen, ash, plastic, and industrial wastes are the primary sources of metals in MSW landfills [42,43]. In contrast, the accumulation of Pb, Cr, Ni, and Zn in soil was found to be lower than the target value standards [34]. However, the quantities of these HMs may be elevated by long-term accumulation [39]. It was reported that HM concentrations were generally lower during the rainy season and higher in dry season [44]. In this study, the sampling stations were close to the leachate canals of the landfill, where the polluted water flowed to and contaminated the surface soil. In contrast, the samples from outside landfill had higher concentrations in the dry season [44]. HM contamination in soils depends on the dose in the MSW and environmental effects [45].

High doses of Cd, Cr, Pb, and Zn were detected in the edible parts (leaves and stems) of *I. aquatica*. Those HMs exceeded the permissible limit of WHO standards (5–86-fold). Although Cr, Cd, and Zn are acknowledged as essential elements to plants, higher concentrations of these HMs can be toxic [46,47,48]. The accumulation of Pb, Cd, and Cr in *I. aquatica* presents health problems for people living in the landfill as well as nearby villagers. This vegetable is commonly collected and sold in local markets and is also used to feed the residents’ animals (pigs). 

It was found that the grass *P. purpureum* absorbed a much higher quantity of Cd as well as other HMs than *I. aquatica* in the landfill (Table 4), perhaps due to the deeper roots of the grass grown in soils, which elevates its ability to absorb heavy metals [49]. Several plants, such as *Pistia stratiotes*, *Eichhornia crassipess*, *Hydrocotyle umbellatta*, *Lemna minor*, *Tyhpa latifolia*, *Scirpus acutus* [50], *Micranthemum ubrosum* [51], and *I. aquatica* [52], play a significant role for phytoremediation, as they are one of the best and cheapest cleanup technologies for contaminated soils, groundwater, and wastewater. However, the high contamination of HMs in *P. purpureum* apparently affected the health of cattle such as cows and buffalos, something which requires further investigation. People living near the MSW landfill in Vientiane may be exposed to HM toxicity at high levels, which would probably lead to health effects [8,53]. Although some metals are essential for biological systems in humans and animals, acting as structural and catalytic components of proteins and enzymes, in higher concentrations, they can be toxic [54,55]. The findings of this study highlight the environmental risks posed by HMs in the MSW landfill of Vientiane. 

This study is the first to examine the HM contamination of water, soil, and plants in the MSW landfill in Vientiane, Laos. It provided detailed information on the polluted levels of HMs in underground water, soil, and vegetation in the MSW landfill. The most problematic HMs included Pb, Cr, Cd, Cu, and Zn (Table 2, Table 3 and Table 4) that may seriously affect the health of local residents near the MSW landfill and environment. Similar investigations of MSW landfills in other big cities of Laos, such as Savannakhet, Pakxe, and Thakhek, should be conducted. Analyzing how much HMs from MSW has penetrated into the environment is necessary for health protection in developing countries. In neighboring countries of Laos, such as Thailand and Vietnam, although the analysis of HMs has been well organized [56,57], MSW pollution has been unstoppable due to the mismanagement of waste classifications. The results of this study should be also submitted to the authorities in Vientiane, Laos, as they are relevant to environmental and health protection. Lawmakers should address the level of toxic HM penetration into the water, soil, and plants in the MSW landfill. 

## 5. Conclusions

This study provided evidence of the serious pollution of the water, soils, and plants growing in the MSW landfill in Vientiane, Laos. Except for Ni, the polluted levels of Cd, Cr, Cu, Pb, and Zn exceeded the standards of ANESs, WHO, and Dutch Pollutant Standards, VROM (2000) to reach levels of eco-toxicological risk. The high accumulation of HMs in the edible plant parts of the widely consumed vegetable *I. aquatica* may cause a dilemma for the villagers living in and around the MSW landfill. The leachates by these HMs from MSW to the surrounding area may enlarge the extent of environmental pollution and health problems, thus requiring immediate phytoremediation and settlement. Frequent monitoring of the surface water, groundwater, and soil quality is necessary to determine the pollution levels and possibly initiate remedial measures. Education and legislation on landfill waste management must be academic and strict, from elementary school to university levels, throughout the country. In addition, the government should pay attention to improving landfill systems such as wastewater and leachate treatment systems. The MWS should be classified and analyzed to understand what sources in the MSW landfill principally caused the leaches of HMs in groundwater, soil, and vegetation. This may help to prevent the pollution by HMs from the MWS, therefore reducing the health problems of Laotian residents in the MSW landfill as well as in Vientiane.

## Figures and Tables

**Table 1:** Description of water, soil, and plant sampling sites (inside and outside the landfill).

Site	Latitude	Longitude	Description of Location
Surface water sampling sites			
W1	18°4′50.45″	102°51′13.20″	Leachate and wastewater were runoff from the landfill to the wetland (upstream)
W2	18°4′53.85″	102°51′13.35″	Leachate and wastewater were runoff from the landfill to wetland (middle stream)
W3	18°5′0.20″	102°51′12.26″	Leachate and wastewater were runoff from the landfill to wetland (downstream)
Groundwater sampling sites			
G1	18°4′28.40″	102°51′12.31″	Available groundwater (well) inside landfill used for the landfill management’s office and waste pickers
G2	18°5′3.26″	102°51′11.70″	Available well used for domestic purposes of farmers was outside the landfill about 70 m away
Soil sampling sites			
S1	18°4′48.32″	102°50′50.46″	Random samples of soils in the landfill were near recharge canals
S2	18°4′45.62″	102°50′57.60″	Random samples of soils in the landfill were near recharge canals
S3	18°4′33.17″	102°51′0.81″	Random samples of soils in the landfill were near recharge canals
S4	18°5′2.90″	102°51′12.59″	Random samples of soils were outside the landfill site about 60 m away
Plant sampling sites			
IA1	18°4′46.82″	102°50′55.98″	Random samples of *Ipomoea aquatica* in the wastewater or leachate area (inside landfill)
IA2	18°4′26.91″	102°51′11.23″	Random samples of *I. aquatica* in the fish pond was near the landfill’s office.
P1	18°4′33.17″	102°51′0.81″	Random samples of grass in the landfill
P2	18°5′2.90″	102°51′12.59″	Random samples of grass were outside the landfill site about 70 m away

**Table 2:** Heavy metal concentrations between the wet and dry seasons in surface and groundwater (mg/L).

Site	Heavy Metal	Wet Season	Dry Season	Standards (mg/L)	
		Mean ± SD	Mean ± SD	ANESs	WHO
W1-3	Cd	ND	ND	0.03	-
	Cr	0.05 ± 0.015 ^b^	0.19 ± 0.057 ^a^	0.5	-
	Cu	0.01 ± 0.005 ^b^	0.05 ± 0.018 ^a^	0.5	-
	Ni	0.01 ± 0.001 ^b^	0.04 ± 0.005 ^a^	0.2	-
	Pb	0.02 ± 0.008 ^b^	0.17 ± 0.042 ^a^	0.2	-
	Zn	ND	ND	1.0	-
G1	Cd	ND	ND	0.003	0.003
	Cr	0.06 ± 0.01 ^a^	0.08 ± 0.020 ^a^	0.05	0.05
	Cu	0.01 ± 0.011 ^a^	0.004 ± 0.01 ^a^	1.50	2.00
	Ni	0.01 ± 0.010	ND	0.02	0.07
	Pb	0.05 ± 0.020 ^a^	0.06 ± 0.013 ^a^	0.01	0.01
	Zn	ND	ND	5.00	3.00
G2	Cd	ND	ND	0.003	0.003
	Cr	0.02 ± 0.019 ^a^	0.04 ± 0.022 ^a^	0.05	0.05
	Cu	ND	ND	1.50	2.00
	Ni	0.001 ± 0.001 ^b^	ND	0.02	0.07
	Pb	ND	0.04 ± 0.014 ^a^	0.01	0.01
	Zn	ND	ND	5.00	3.00

SD: standard deviation; ND: not detected; W: station of surface water sampling, including three main stations/points (W1–3); G1: groundwater in the landfill; G2: groundwater outside the landfill; WHO: World Health Organization, Guidelines for Drinking-Water Quality (2011); ANESs: Agreement on the National Environmental Standards of Laos (2009); values in a row with similar superscript letters are not significantly different (*p* < 0.05).

**Table 3:** Accumulation of heavy metals in soils compared with the Dutch standards (mg/kg).

Sites	Heavy Metal	Wet Season	Dry Season	Dutch Standards	
		Mean ± SD	Mean ± SD	Tv	Iv
S1–3	Cd	3.76 ± 0.33 ^a^	3.73 ± 1.12 ^a^	0.8	12.0
	Cr	39.67 ± 3.78 ^a^	48.08 ± 13.67 ^a^	100.0	380.0
	Cu	66.82 ± 27.52 ^a^	54.06 ± 20.99 ^a^	36.0	190.0
	Ni	19.43 ± 0.84 ^a^	19.94 ± 4.91 ^a^	35.0	210.0
	Pb	80.17 ± 19.33 ^a^	67.99 ± 19.07 ^a^	85.0	530.0
	Zn	77.46 ± 57.88 ^a^	52.48 ± 34.59 ^a^	140.0	720.0
S4	Cd	1.02 ± 0.64 ^b^	1.06 ± 0.05 ^b^	0.8	12.0
	Cr	10.02 ± 4.24 ^b^	19.33 ± 1.95 ^b^	100.0	380.0
	Cu	7.96 ± 2.79 ^b^	11.70 ± 0.50 ^b^	36.0	190.0
	Ni	5.65 ± 1.72 ^b^	9.61 ± 0.06 ^b^	35.0	210.0
	Pb	16.03 ± 5.40 ^b^	21.47 ± 0.42 ^b^	85.0	530.0
	Zn	4.39 ± 2.68 ^b^	4.79 ± 0.56 ^b^	140.0	720.0

SD: standard deviation; S: stations of soil sampling in landfill, including three main stations (S1–3); S4: the main station of soil sampling outside the landfill; Tv: target value (values > Tv: eco-toxicological risk); Iv: intervention value (values > Iv: environmental risk); values in columns with similar superscript letters are not significantly different (*p* < 0.05).

**Table 4:** Levels of heavy metals (mg/kg) in plants between the wet and dry seasons.

Plant Name/Site	Samples	Cd	Cr	Cu	Ni	Pb	Zn
	**Wet Season**						
*I. aquatica*	Leaves	ND	17.00 ± 4.27 ^a^	8.30 ± 5.12 ^a^	0.24 ± 0.34 ^a^	13.26 ± 7.06 ^a^	1.54 ± 0.64 ^a^
	Stem	ND	18.63 ± 6.10 ^a^	9.63 ± 9.75 ^a^	ND	7.48 ± 7.11 ^a^	8.54 ± 11.97 ^a^
	Roots	0.16 ± 0.21 ^a^	26.62 ± 1.54 ^a^	36.79 ± 13.32 ^a^	11.01 ± 9.74 ^a^	38.94 ± 15.85 ^a^	26.27 ± 6.46 ^a^
Grass/P1	Leaves	ND	11.32 ± 2.05 ^b^	21.10 ± 2.36 ^b^	0.25 ± 0.24 ^b^	2.85 ± 2.69 ^b^	4.27 ± 7.39 ^c^
	Stems	3.07 ± 1.57 ^b^	40.47 ± 25.75 ^b^	55.50 ± 37.50 ^b^	15.93 ± 13.81 ^b^	47.30 ± 36.84 ^b^	38.47 ± 6.12 ^ab^
	Roots	8.24 ± 3.13 ^a^	164.33 ± 50 ^a^	193.7 ± 30.02 ^a^	71.33 ± 22.08 ^a^	181 ± 49 ^a^	55.03 ± 8.09 ^a^
Grass/P2 WHO standards	Leaves	ND	11.14 ± 2.75 ^b^	21 ± 14.53 ^b^	0.55 ± 0.08 ^b^	3.31 ± 2.97 ^b^	0.33 ± 0.57 ^c^
	Stems	ND	12.03 ± 2.29 ^b^	63.2 ± 17.34 ^b^	0.08 ± 0.14 ^b^	2.83 ± 2.72 ^b^	15.86 ± 11.12 ^bc^
	Roots	1.32 ± 1.72 ^b^	20.22 ± 24.25 ^b^	29.97 ± 34.42 ^b^	5.13 ± 8.89 ^b^	21.07 ± 26.92 ^b^	35.27 ± 16.64 ^ab^
		0.02	1.30	10.00	10.00	2.00	0.60
	**Dry Season**						
*I. aquatica*	Leaves	ND	15.37 ± 1.27 ^a^	21.60 ± 7.68 ^a^	0.39 ± 0.29 ^a^	9.66 ± 1.64 ^a^	27.60 ± 24.00 ^a^
	Stems	0.11 ± 0.15 ^a^	16.68 ± 5.44 ^a^	35.98 ± 17.04 ^a^	0.65 ± 0.30 ^a^	9.12 ± 2.55 ^a^	8.25 ± 3.02 ^a^
	Roots	1.12 ± 1.16 ^a^	26.10 ± 5.94 ^a^	49.23 ± 0.47 ^a^	5.79 ± 3.13 ^a^	34.66 ± 4.05 ^a^	29.18 ± 24.45 ^a^
Grass/P1	Leaves	0.96 ± 0.93 ^a^	35.37 ± 4.65 ^a^	218.7 ± 9.07 ^a^	2.45 ± 0.85 ^a^	16.27 ± 3.04 ^a^	37.8 ± 23.19 ^a^
	Stems	0.07 ± 0.13 ^a^	7.52 ± 2.56 ^c^	8.42 ± 0.5 ^d^	0.40 ± 0.70 ^a^	0.38 ± 0.66 ^b^	8.52 ± 9.59 ^a^
	Roots	2.07 ± 1.83 ^a^	18.7 ± 4.06 ^b^	48 ± 13.42 ^c^	4.57 ± 1.91 ^a^	8.91 ± 3.85 ^ab^	33.1 ± 23.31 ^a^
Grass/P2 WHO standards	Leaves	0.63 ± 0.60 ^a^	42.47 ± 4.61 ^a^	159 ± 6.24 ^b^	1.9 ± 2.11 ^a^	13.36 ± 7.58 ^a^	22.9 ± 31.02 ^a^
	Stems	0.09 ± 0.15 ^a^	7.07 ± 2.74 ^e^	7.55 ± 2.44 ^d^	0.72 ± 1.25 ^a^	0.25 ± 0.27 ^b^	7.07 ± 12.24 ^a^
	Roots	1.05 ± 1.82 ^a^	13.5 ± 2.98 ^be^	39.4 ± 18.71 ^c^	2.37 ± 2.64 ^a^	5.37 ± 6.21 ^ab^	17.7 ± 24.37 ^a^
		0.02	1.30	10.00	10.00	2.00	0.60

Values are mean ± SD (standard error); WHO: World Health Organization; P1 and P2: sampling stations of *Pennisetum purpureum* in and outside the landfill, respectively; ND: not detected. Columns with similar superscript letters are not significantly different at *p* < 0.05. P1 and P2: locations of sampling in and outside the landfill, respectively.

## References

[1] United Nations Environment Programme (UNEP) (2017). Waste Management in ASEAN Countries. https://wedocs.unep.org/bitstream/handle/20.500.11822/21134/waste_mgt_asean_summary.pdf?sequence=1&isAllowed=y.

[2] Bakis R., Tuncan A. (2011). An investigation of heavy metal and migration through groundwater from the landfill area of Eskisehir in Turkey. Environ. Monit. Assess..

[3] Giusti L. (2009). A review of waste management practices and their impact on human health. Waste Manag..

[4] Esakku S., Palanivelu K., Joseph K. Assessment of heavy metals in a municipal solid waste dumpsite. Proceedings of the Workshop on Sustainable Landfill Management.

[5] Kanmani S., Gandhimathi R. (2012). Assessment of heavy metal contamination in soil due to leachate migration from an open dumping site. Appl. Water Sci..

[6] Slack R.J., Gronow J.R., Voulvoulis N. (2005). Household hazardous waste in municipal landfills: Contaminants in leachate. Sci. Total Environ..

[7] Chary N.S., Kamala C., Raj D.S. (2008). Assessing risk of heavy metals from consuming food grown on sewage irrigated soils and food chain transfer. Ecotoxicol. Environ. Safety.

[8] Nica D.V., Bura M., Gergen I., Harmanescu M., Bordean D. (2012). Bioaccumulative and conchological assessment of heavy metal transfer in a soil-plant-snail food chain. Chem. Cent. J..

[9] Jaishankar M., Tseten T., Anbalagan N., Mathew B.B., Beeregowda K.N. (2014). Toxicity, mechanism and health effects of some heavy metals. Interdisciplinary Toxicol..

[10] Muddarisna N., Krisnayanti B.D., Utami S.R., Utami E., Handayanto E. (2013). Phytoremediation of mecury-contaminated soil using three wild plant species and its effect on maize growth. Applied Ecol. Environ. Sci..

[11] Arora M., Kiran B., Rani S., Rani A., Kaur B., Mittal N. (2008). Heavy metal accumulation in vegetables irrigated with water from different sources. Food Chem..

[12] Chen L., Zhou S., Shi Y., Wang C., Li B., Li Y., Wu S. (2018). Heavy metals in food crops, soil, and water in the Lihe River Watershed of the Taihu Region and their potential health risks when ingested. Sci. Total Environ..

[13] Mertz W. (1981). The essential trace elements. Science.

[14] Muchuweti M., Birkett J., Chinyanga E., Zvauya R., Scrimshaw M., Lester J. (2006). Heavy metal content of vegetables irrigated with mixtures of wastewater and sewage sludge in Zimbabwe: Implications for human health. Agric. Ecosyst. Environ..

[15] Tchounwou P.B., Yedjou C.G., Patlolla A.K., Sutton D.J. (2012). Heavy metals toxicity and the environment. Molecular, Clinical and Environmental Toxicology.

[16] Öman C.B., Junestedt C. (2008). Chemical characterization of landfill leachates-400 parameters and compounds. Waste Manag..

[17] Clarke B.O., Anumol T., Barlaz M., Snyder S.A. (2015). Investigating landfill leachate as a source of trace organic pollutants. Chemosphere.

[18] Kabata-Pendias A., Pendias H. (2010). Trace Elements in Soils and Plants.

[19] Hinchman R.R., Negri M.C., Gatliff E.G. Phytoremediation: Using green plants to clean up contaminated soil, groundwater and wastewater. Proceedings of the International Topical Meeting on Nuclear and Hazardous Waste Management.

[20] Kamal M., Ghaly A.E., Mahmoud N., Cote R. (2004). Phytoaccumulation of heavy metals by aquatic plants. Environ. Int..

[21] Kivaisi A.K. (2001). The potential for constructed wetlands for wastewater treatment and reuse in developing countries: A review. Ecol. Eng..

[22] Madera-Parra C.A., Peña-Salamanca E.J., Peña M.R., Rousseau D.P., Lens P.N. (2014). Phytoremediation of landfill leachate with *Colocasia esculenta*, *Gynerum sagittatumand*, and *Heliconia psittacorumin* constructed wetlands. Int. J. Phytoremediat..

[23] Climate and Clean Air Coalition Municipal Solid Waste Initiative (CCAC) (2015). Solid Waste Management City Profile, Vientiane Capital, LAO People’s Democratic Republic. http://www.waste.ccacoalition.org/sites/default/files/files/vientiane_city_profile_vientiane_capital_lao.pdf.

[24] Vodyanitskii Y.N. (2016). Biochemical processes in soil and groundwater contaminated by leachates from municipal landfills (mini review). Ann. Agrar. Sci..

[25] Wuana R.A., Okieimen F.E. (2011). Heavy metals in contaminated soils: A review of sources, chemistry, risks and best available strategies for remediation. Isrn Ecol..

[26] Ontario Ministry of the Environment and Climate Change (MOECC) (2016). Protocol for the Sampling and Analysis of Industrial/Municipal Wastewater. http://www.downloads.ene.gov.on.ca/envision/env_reg/er/documents/2016/011–7834_Protocol.pdf.

[27] Government of Western Australia (2009). Field Sampling Guideline: A Guideline for Field Sampling for Surface Water Quality Monitoring Programs. https://www.water.wa.gov.au/__data/assets/pdf_file/0020/2936/87154.pdf.

[28] Gworek B., Dmuchowski W., Koda E., Marecka M., Baczewska A.H., Brągoszewska P., Osiński P. (2016). Impact of the municipal solid waste Łubna landfill on environmental pollution by heavy metals. Water.

[29] SHIMADZU (2005). Environmental Analyses-Shimadzu Analysis Guidebook. https://applicationstation.ssi.shimadzu.com/sites/default/files/environmental-analyses-guidebook.pdf.

[30] Andersen K.J., Kisser M.I. (2004). Digestion of Solid Matrices–Desk Study Horizontal.

[31] Ministry of Natural Resources and Environment, Laos, MONEL (2009). Agreement on National Environmental Standards of Laos (ANESs).

[32] WHO (World Health Organization) (2011). Guidelines for Drinking-Water Quality.

[33] WHO (World Health Organization) (1996). Permissible Limits of Heavy Metals in Soil and Plants.

[34] Bird G., Brewer P.A., Macklin M.G., Balteanu D., Driga B., Serban M., Zaharia S. (2003). The solid-state partitioning of contaminant metals and as in river channel sediments of the mining affected Tisa drainage basin, northwestern Romania and eastern Hungary. Appl. Geochem..

[35] Van Ryan Kristopher R.G., Parilla R. (2014). Analysis of heavy metals in Cebu city sanitary landfill, Philippines. J. Environ. Sci. Manag..

[36] Kar D., Sur P., Mandai S.K., Saha T., Kole R.K. (2008). Assessment of heavy metal pollution in surface water. Int. J. Environ. Sci. Tech..

[37] Engin M.S., Uyanik A., Kutbay H.G. (2015). Accumulation of heavy metals in water, sediments and wetland plants of Kizilirmak Delta (Samsun, Turkey). Int. J. Phytoremediat..

[38] Tatsi A.A., Zouboulis A.I. (2002). A field investigation of the quantity and quality of leachate from a municipal solid waste landfill in a Mediterranean climate (Thessaloniki, Greece). Adv. Environ. Res..

[39] Kjeldsen P., Barlaz M.A., Rooker A.P., Baun A., Ledin A., Christensen T.H. (2002). Present and long-term composition of MSW landfill leachate: A review. Crit. Rev. Eniron. Sci. Tech..

[40] Arao T., Ishikawa S., Murakami M., Abe K., Maejima Y., Makino T. (2010). Heavy metal contamination of agricultural soil and countermeasures in Japan. Paddy Water Environ..

[41] Calace N., Liberatori A., Petronio B.M., Pietroletti M. (2001). Characteristics of different molecular weight fractions of organic matter in landfill leachate and their role in soil sorption of heavy metals. Environ. Pollut..

[42] Awasthi A.K., Zeng X., Li J. (2016). Environmental pollution of electronic waste recycling in India: A critical review. Environ. Pollut..

[43] Long Y.Y., Shen D.S., Wang H.T., Lu W.J., Zhao Y. (2011). Heavy metal source analysis in municipal solid waste (MSW): Case study on Cu and Zn. J. Hazard. Mater..

[44] Olafisoye O.B., Adefioye T., Osibote O.A. (2013). Heavy metals contamination of water, soil, and plants around an electronic waste dumpsite. Environ. Study.

[45] Samadder S.R., Prabhakar R., Khan D., Kishan D., Chauhan M.S. (2017). Analysis of the contaminants released from municipal solid waste landfill site: A case study. Sci. Total Environ..

[46] Cakmak I., Marschner H. (1993). Effect of zinc nutritional status on activities of superoxide radical and hydrogen peroxide scavenging enzymes in bean leaves. Plant Nutrition-from Genetic Engineering to Field Practice.

[47] Athar M., Vohora S.B. (1995). Heavy Metals and Environment.

[48] Rai U.N., Sinha S. (2001). Distribution of metals in aquatic edible plants: *Trapa natans* (Roxb.) Makino and *Ipomoea aquatica* Forsk. Environ. Monit. Assess..

[49] Durowoju O.S., Odiyo J.O., Ekosse G.I.E. (2016). Variations of heavy metals from geothermal spring to surrounding soil and *Mangifera indica*–Siloam village, Limpopo province. Sustainability.

[50] Farid M., Irshad M., Fawad M., Ali Z., Eneji A.E., Aurangzeb N., Ali B. (2014). Effect of cyclic phytoremediation with different wetland plants on municipal wastewater. Int. J. Phytoremediat..

[51] Islam M.S., Ueno Y., Sikder M.T., Kurasaki M. (2013). Phytofiltration of arsenic and cadmium from the water environmnt using *Micranthemum umbrosum* (JF Gmel) SF Blake as a hyperaccumulator. Int. J. Phytoremediat..

[52] Chanu L.B., Gupta A. (2016). Phytoremediation of lead using *Ipomoea aquatica* Forsk. in hydroponic solution. Chemosphere.

[53] Gbaruko B.C., Friday O.V. (2007). Bioaccumulation of heavy metals in some fauna and flora. Int. J. Environ. Sci. Tech..

[54] Liu J., Cao L., Dou S. (2017). Bioaccumulation of heavy metals and health risk assessment in three benthic bivalves along the coast of Laizhou Bay, China. Mar. Pollut. Bull..

[55] Nazir R., Khan M., Masab M., Rehman H.U., Rauf N.U., Shahab S., Shaheen Z. (2015). Accumulation of heavy metals (Ni, Cu, Cd, Cr, Pb, Zn, Fe) in the soil, water and plants and analysis of physico-chemical parameters of soil and water collected from Tanda Dam Kohat. J. Pharm. Sci. Res..

[56] Schneider P., Anh L.H., Wagner J., Reichenbach J., Hebner A. (2017). Solid waste management in Ho Chi Minh City, Vietnam: Moving towards a circular economy?. Sustainability.

[57] Yukalang N., Clarke B., Ross K. (2017). Barriers to effective municipal solid waste management in a rapidly urbanizing area in Thailand. Int. J. Environ. Res. Public Health.

